# The cytotoxic activities of 7-isopentenyloxycoumarin on 5637 cells via induction of apoptosis and cell cycle arrest in G2/M stage

**DOI:** 10.1186/2008-2231-22-3

**Published:** 2014-01-06

**Authors:** Fereshteh Haghighi, Maryam M Matin, Ahmad Reza Bahrami, Mehrdad Iranshahi, Fatemeh B Rassouli, Azadeh Haghighitalab

**Affiliations:** 1 Department of Biology, Faculty of Science, Ferdowsi University of Mashhad, Mashhad, Iran; 2 Cell and Molecular Biotechnology Research Group, Institute of Biotechnology, Ferdowsi University of Mashhad, Mashhad, Iran; 3 Biotechnology Research Center and School of Pharmacy, Mashhad University of Medical Sciences, Mashhad, Iran

**Keywords:** Bladder cancer, Cytotoxicity, 7-isopentenyloxycoumarin, Apoptosis, Cell cycle

## Abstract

**Background:**

Bladder cancer is the second common malignancy of genitourinary tract, and transitional cell carcinomas (TCCs) account for 90% of all bladder cancers. Due to acquired resistance of TCC cells to a wide range of chemotherapeutic agents, there is always a need for search on new compounds for treatment of these cancers. Coumarins represent a group of natural compounds, which some of them have exerted valuable anti-tumor activities. The current study was designed to evaluate anti-tumor properties and mechanism of action of 7-isopentenyloxycoumarin, a prenyloxycoumarin, on 5637 cells (a TCC cell line).

**Results:**

MTT results revealed that the cytotoxic effects of 7-isopentenyloxycoumarin on 5637 cancerous cells were more prominent in comparison to HDF-1 normal cells. This coumarin increased the amount of chromatin condensation and DNA damage in 5637 cells by 58 and 33%, respectively. The results also indicated that it can induce apoptosis most probably via activation of caspase-3 in these cells. Moreover, propidium iodide staining revealed that 7-isopentenyloxycoumarin induced cell cycle arrest at G2/M stage, after 24 h of treatment.

**Conclusion:**

Our results indicated that 7-isopentenyloxycoumarin had selective toxic effects on this bladder cancer cell line and promoted its effects by apoptosis induction and cell cycle arrest. This coumarin can be considered for further studies to reveal its exact mechanism of action and also its anti-cancer effects *in vivo*.

## Background

About 7 million people die from different types of cancer, which makes it the second cause of death worldwide every year [1]. Bladder cancer is the second most common genitourinary malignancy with a higher incidence in men. More than 90% of all bladder cancers are transitional cell carcinomas (TCCs). Although cisplatin-based combination chemotherapies like MVAC (methotrexate-vinblastine-adriamycin-cisplatin) are the standard treatments in patients with TCC, harnessing metastatic bladder cancer still remains one of the main challenges in urologic oncology [2,3]. The resistance of TCC cells to a wide range of chemotherapeutic agents is the main reason for the failure of such treatments [4].

Apoptosis, also known as programmed cell death, is a naturally occurring process that plays a central role in the normal development and homeostasis of all multi-cellular organisms [5]. Apoptosis is accompanied by cell shrinkage, reduction of cellular volume, nuclear fragmentation, chromatin condensation, plasma membrane blebbing and engulfment by phagocytes *in vivo*[6]. There are two main apoptotic pathways in mammals: the extrinsic or death receptor pathway and the intrinsic or mitochondrial pathway. Both mechanisms end at the point of execution phase, which is initiated by the cleavage of executioner caspases and results in the apoptotic morphology. Caspase-3 is one of the most important executioner caspases, which is activated in both extrinsic and intrinsic pathways [7].

Coumarins are a large group of natural compounds that are mainly found in the plant families of Rutaceae, Apiaceae and Umbelliferae as well as some bacteria and fungi [8]. Biological properties of coumarins include anti-coagulant, anti-viral, anti-microbial, anti-inflammatory, anti-oxidant and anti-tumor activities [9-12]. Induction of cell cycle arrest by coumarins is also reported frequently [9-11]. Prenyloxycoumarins represent a class of secondary metabolites that based on the length of their carbon chains, are categorized in three groups; C5 (isopentenyl), C10 (geranyl) and C15 (linear, mono- or bicyclic sesquiterpenyl) [8]. One of these studied coumarins is 7-isopentenyloxycoumarin (7-IP) [13].

7-IP, a secondary metabolite with an isopentenyl chain fixed at C7 of the 1, 2-benzopyrone ring, exerts valuable and promising anti-microbial, anti-inflammatory and anti-cancer effects [14]. The chemopreventive [15], neuroprotective [16] and anti-genotoxic [17] properties of 7-IP have been shown previously. This coumarin was first isolated by Prokopenko from the fruit of *Libanotis intermedia* in 1966. In nature, 7-IP is biosynthesized from 7-hydroxycoumarin and dimethylallyl diphosphate [14] and is widespread in edible vegetables and fruits such as grapefruit, lemon, orange, mandarin and many other plants [18].

In this study, 7-IP was synthesized by a reaction between 7-hydroxycoumarin and its relevant prenyl bromide. Here, we evaluated its toxic effects on 5637 and HDF-1 cell lines by MTT assay. Since, there is no report on the mechanism of action of 7-IP, we report the effects of this coumarin in more details by DAPI staining, comet assay, caspase-3 activity and cell cycle analysis.

## Methods

### Chemical synthesis

**Figure 1:**
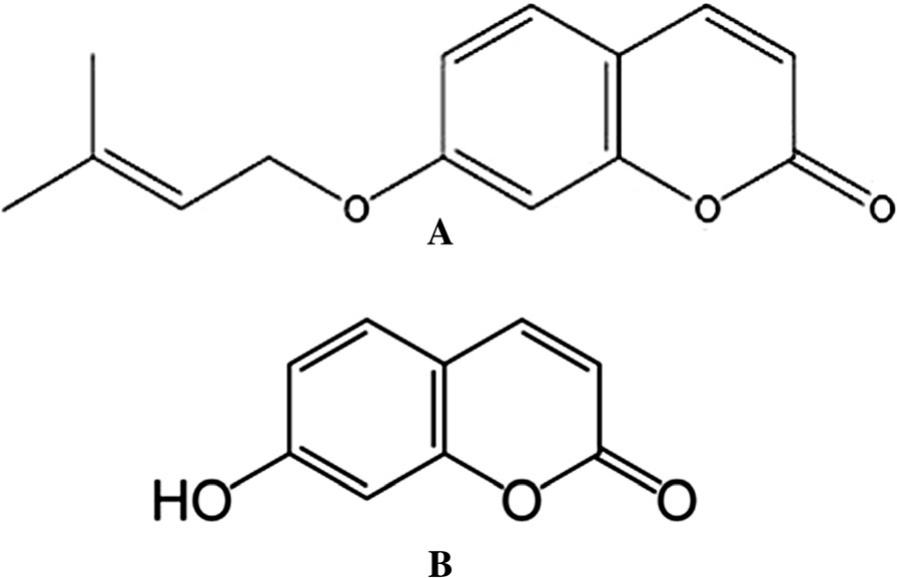
**Chemical structure of coumarins. A)** 7-IP and **B)** 7-hydroxycoumarin.

7-IP (Figure 1A) was synthesized as described by Askari *et al.*[13]. Briefly, it was synthesized by a reaction between 7-hydroxycoumarin (Figure 1B) and isopentenyl bromide in the presence of DBU (1, 8-diazabicyclo [5.4.0]undec-7-ene), in acetone at room temperature. The purity and chemical structure of 7-IP was then assessed by ^1^H- and ^13^C-NMR spectroscopy (Additional file 1: Table S1 and Additional file 2: Table S2).

### Preparation of different 7-IP concentrations

To prepare different concentrations of 7-IP (10 to 100 μg/ml), 2 mg of the powder was dissolved in 500 μl dimethyl sulfoxide (DMSO, Merck, Germany), and diluted with complete culture medium before experiments. Since the solvent has cytotoxic effects, the control treatments were run at the same time with equivalent solutions of DMSO.

### Cell lines and cell culture

Human 5637 cells (a TCC subline) were obtained from Pasteur Institute (Tehran, Iran). These epithelial like cells were first derived in 1974 of a primary bladder carcinoma from a 68- year old Caucasian man. HDF-1 (human dermal fibroblast) cells, derived from human skin fibroblasts, were a generous gift from Royan Institute (Tehran, Iran). All cells were maintained in Dulbecco’s modified Eagle’s medium (DMEM, Gibco, Scotland) supplemented with 10% heat-inactivated fetal bovine serum (FBS, Gibco, Scotland) for 5367 cells, and 15% FBS for HDF-1 cells. 5637 and HDF-1 cells were grown at 37°C in a humisdified atmosphere of 10% and 5% CO_2_ in air, respectively. To subculture the cells, they were washed with phosphate buffered saline (PBS) and incubated with 0.25% trypsin/1 mM ethylenediaminetetraacetic acid (EDTA) (Gibco, Scotland) for 5 min. Detached cells were resuspended in fresh serum-containing medium to inactivate the trypsin and transferred to new labeled flasks.

### Cell viability assay

Cell viability was determined by MTT assay [19]. In this method, tetrazolium dye, 3-(4,5-Dimethyl-2-thiazolyl)-2,5-diphenyl-2*H*-tetrazolium bromide, is reduced by mitochondrial enzymes to insoluble purple formazan in living cells. The absorbance of the colored product (after dissolving in DMSO) can be determined by a spectrophotometer. Briefly, 5637 and HDF-1 cells were seeded at a density of 1.3 × 10^4^ and 1.0 × 10^4^ cells/well in 96-well tissue culture plates (Orange Scientific, France), respectively. The cells were allowed to attach and grow for 24 h. In order to identify the half maximal inhibitory concentration (IC_50_) values of 7-IP, cells were treated with increasing concentrations (10 to 100 μg/ml) of the compound for 24, 48 and 72 h. To perform MTT assay, 5 mg MTT dye (Sigma Aldrich, Germany) was dissolved in 1 ml PBS, filtered through a 0.22 μm filter (Orange Scientific, France) and used freshly before each experiment. Then 20 μl MTT solution (500 μg/ml final concentration) was added to each well and plates were incubated at 37°C for 4 h. After this period, the remaining MTT solution was removed and 150 μl DMSO was added to each well to dissolve the formazan crystals. The absorbance of each well was then measured at 545 nm using an enzyme linked immunosorbent assay (ELISA) plate reader (Awareness, USA). The percentage of cell viability was calculated by dividing the mean absorbance of each treatment (7-IP) to the mean absorbance of its controls (DMSO) multiply by 100.

### Morphological alterations

Cells treated with various concentrations of 7-IP were observed under a light inverted microscope (Olympus, Japan) for morphological changes after 24, 48 and 72 h of the treatments.

### Apoptosis assay with DAPI staining

In order to evaluate the apoptotic effects of 7-IP semi-quantitatively, DAPI (4′, 6-diamidino-2-phenylindole dichloride) staining was performed. To do so, 5637 cells were grown to 80% confluency in 6-well tissue culture plates (Orange Scientific, France). Then the cells were treated with 65 μg/ml 7-IP and its equivalent amount of DMSO (1.625%) for 72 h. Cells were then washed gently with PBS and fixed with 4% paraformaldehyde (Sigma, Germany) for 10 min at room temperature. After that cells were washed with PBS, permeabilized with 0.1% Triton X-100 (Merck, Germany) and stained with 2 μg/ml DAPI (Merck, Germany) at 37°C for 10 min. Approximately, 700 cells from each treatment were visualized and counted under a fluorescent microscope (Olympus, Japan). Chromatin condensation was considered as a specific criterion for apoptotic morphology.

### Alkaline comet assay

To investigate the DNA-damaging effects of 7-IP, the alkaline version of comet assay, which is a rapid, reliable and quantitative technique for detection of possible DNA lesions at the individual eukaryotic cells, was performed [20]. Briefly, untreated 5637 and HDF-1 cells, and cells treated for 72 h with 65 μg/ml 7-IP and its DMSO control were trypsinized and centrifuged at 1100 g for 10 min. The cell pellets were resuspended in 50 μl PBS and then mixed with 50 μl of 1.5% (w/v) low melting point agarose (LMA, Fermentas, Germany) and spread on glass slides precoated with 1% (w/v) normal melting point agarose (NMA, Helicon, Russia). Four slides were prepared for each treatment. The slides were kept for 20 min at 4°C to allow the agarose to solidify. After 20 min 100 μl of 0.75% (w/v) LMA was added to each slide, and kept for another 20 min at 4°C. When the gel was solidified, slides were submerged in freshly prepared lysing buffer (2.5 M NaCl, 100 mM Na_2_EDTA, 10 mM Tris, 2% (v/v) Triton X-100, pH 10) for at least 4 h at 4°C. Then the slides were placed in an electrophoresis chamber filled with freshly prepared cold alkaline electrophoresis buffer (1 mM EDTA, 0.3 N NaOH, pH 13) and incubated for 30 min at 4°C. Electrophoresis was conducted at 300 mA and 25 V for 20 min at 4°C. Slides were then washed with ice-cold neutralizing buffer (0.4 M Tris–HCl, pH 7.5) for three times, dried with 96% ethanol and stained with ethidium bromide (20 μg/ml). The slides were viewed and analyzed with a fluorescent microscope (Olympus, Japan). One hundred cells per slide were evaluated and the mean of comet tail (a product of fraction of DNA in tail and tail length) was determined using TriTek Cometscore version 1.5 software. The DNA damage was expressed as % DNA in tail.

### Caspase-3 activity assay

Caspase-3 activity was assessed using caspase-3 colorimetric assay kit (Abcam, USA) according to manufacturer’s protocol. Briefly, 5637 cells were treated with 65 μg/ml of 7-IP and its equivalent amount of DMSO for 12 h. Cells treated with 15 μg/ml of cisplatin for 24 h were used as a positive control. After the period of treatments, cells were trypsinized and centrifuged at 1100 g for 10 min. Cell pellets were suspended in 1 ml cold PBS and centrifuged at 4000 g for 5 min at 4°C. In order to extract total protein, cell pellets were resuspended and lysed with 100 μl of chilled lysis buffer and incubated on ice for 10 min. Cell lysates were then centrifuged at 10000 g for 1 min at 4°C. The concentration of proteins was measured by Bradford assay. Then 50 μl of 2× reaction buffer containing 10 mM DTT and 5 μl of 4 mM caspase-3 substrate (DEVD-*p*NA) were added to 200 μg protein from each sample and incubated at 37°C for 4 h. The *p*-NA light emission was quantified using ELISA plate reader at 405 nm. Comparison of the absorbance of *p*-NA from an apoptotic sample with an uninduced control allowed determination of the fold increase in caspase-3 activity.

### Cell cycle analysis

The cell cycle distribution was assayed by flow cytometry after DNA staining with propidium iodide (PI). 5637 cells were treated with 65 μg/ml 7-IP and its equivalent amount of DMSO. After 24 h of treatments, floated cells were harvested and attached cells were trypsinized, washed with a protein-containing buffer (PBS + 5% FBS) and centrifuged at 1100 g for 10 min. The cell pellets were resuspended in 500 μl PBS containing 5% FBS and centrifuged at 200 g for 7 min at 4°C. After that cells were stained with a solution containing 100 μg/ml PI (Sigma Aldrich, Germany), 0.1% Triton X-100, 0.1% sodium citrate (Merck, Germany) and RNase (100 μg/ml) (Merck, Germany) in ice cold PBS. The samples were then incubated at 4°C in dark for 1 h and flow cytometric analyses were performed using a FACSCalibur (BD Biosciences, USA) instrument and WinMDI version 2.9 software.

### Statistical analyses

Data generated from experiments were collected in a completely randomized design. All assays were carried out at least three times. Statistical procedures were performed using Microsoft Office Excel (2007) SPSS, version 16.0 (SPSS software Inc., Chicago, IL, USA) and SAS, version 9.1 (SAS Institute, Inc., Cary, N.C., USA) softwares. A one-way analysis of variance (ANOVA) according to general linear model procedure of SAS software was performed to evaluate possible differences among the treatments. The statistical significant differences among means between each group were determined by using Tukey’s single-step multiple comparison test. Differences among treatment means were compared at *P*-value of < 0.001. Values are expressed as mean ± SD.

## Results

### IC_50_ value determination of 7-IP in 5637 and HDF-1 cell lines

**Figure 2:**
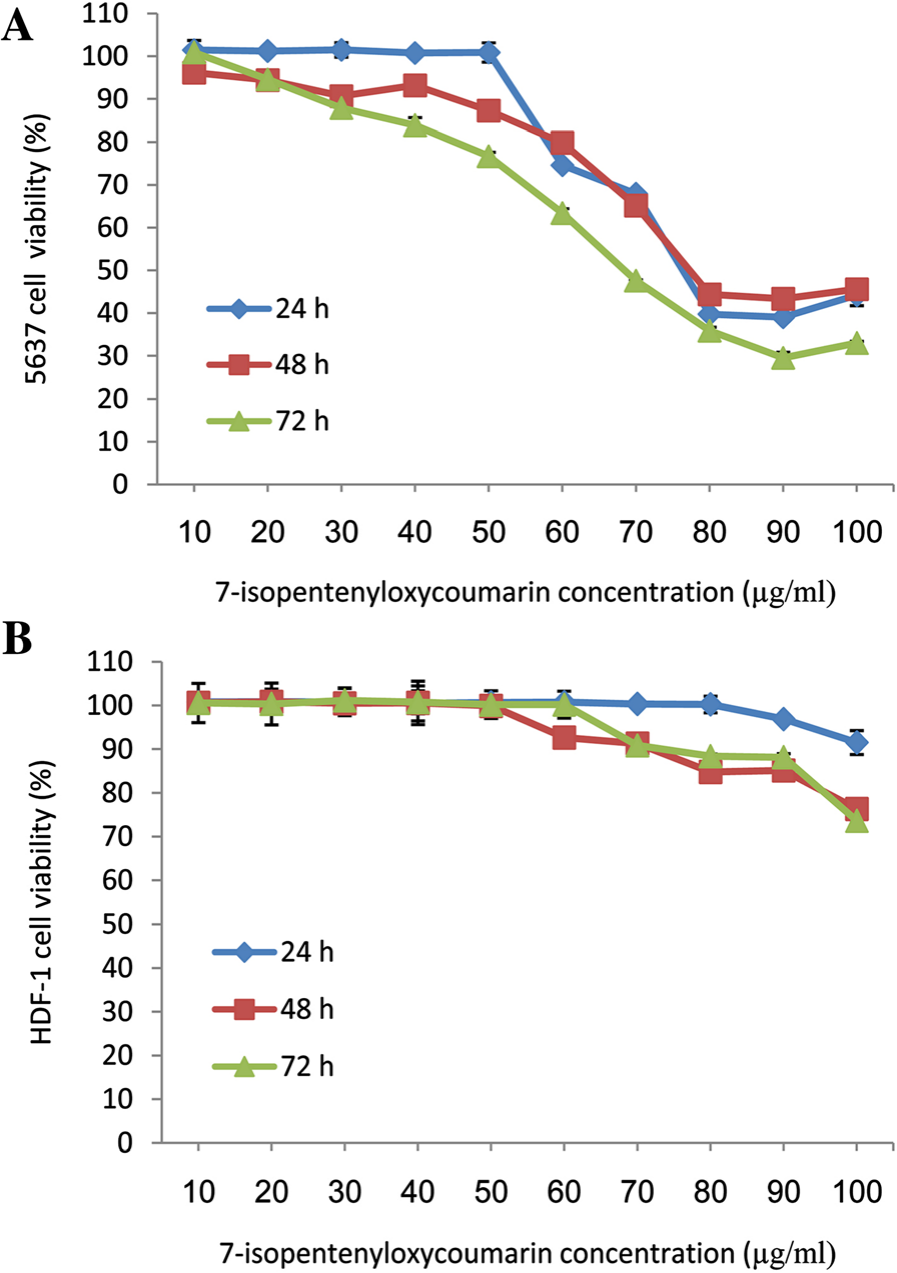
**Time-based dose response curves of cancerous and normal cells.** 5637 **(A)** and HDF-1 **(B)** cells treated with 7-IP during 24, 48 and 72 h. All the points represent results from three independent experiments performed in triplicate. Data are expressed as mean ± SD.

The cytotoxic effects of 7-IP were assessed by MTT assay in 5637 and HDF-1 cells exposed to 10–100 μg/ml of this coumarin for three consecutive days. Cell survival analyses showed that 7-IP had a concentration and time-dependent inhibitory effect on the growth of 5637 cells (Figure 2A). By increasing the concentrations, cell viabilities were significantly different (*P* < 0.001) especially after 48 and 72 h of treatments. The IC_50_ values of this coumarin were calculated as 76, 76 and 65 μg/ml after 24, 48 and 72 h of treatments, respectively.

To assess the cytotoxic effects of 7-IP on normal cells, HDF-1 cells were used. As shown in Figure 2B, although this coumarin induced cell death in these non-cancerous cells, but its cytotoxic effects were not significant in comparison to 5637 cells. Statistical analysis revealed that the slight cytotoxic effects of 7-IP on HDF-1 cells were not dose-dependent except at higher concentrations.

### Morphological alterations

**Figure 3:**
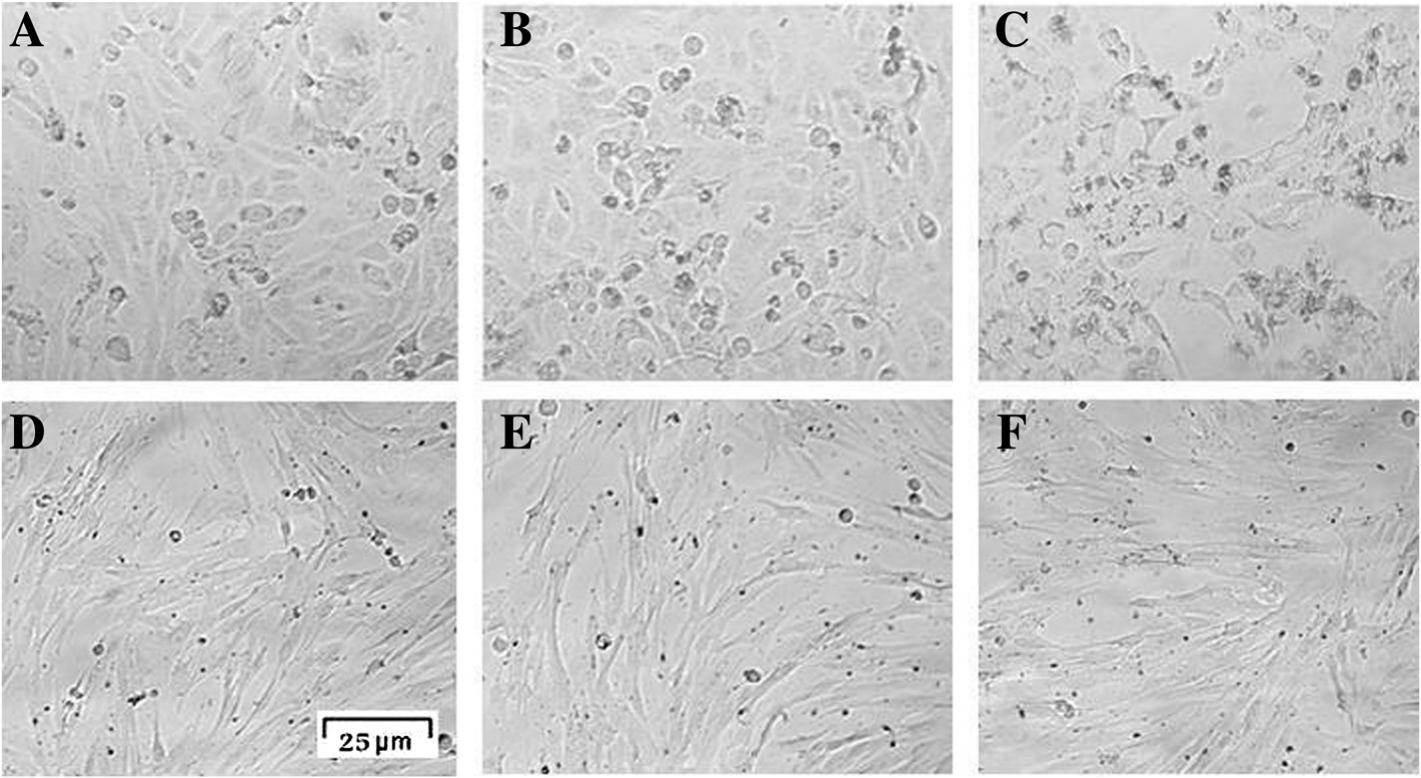
**Light microscopic images of 5637 and HDF-1 cells.** Untreated 5637 cells **(A)**. 5637 cells treated with 1.625% DMSO **(B)** represent little morphological changes as compared with untreated cells. 5637 cells treated with 65 μg/ml 7-IP **(C)** revealed prominent cytoplasmic granulation and cell death. Untreated HDF-1 cells **(D)**. HDF-1 cells treated with 2.5% DMSO **(E)**. HDF-1 cells treated with 100 μg/ml 7-IP after 72 h with little changes in their morphology **(F)**.

The cytotoxic effects of 7-IP were confirmed by morphological observations. As shown in Figure 3 (A-C), 5637 cells treated with 65 μg/ml 7-IP for 72 h, revealed prominent cytoplasmic granulation and cell number was significantly decreased as compared with control and untreated cultures. In the case of HDF-1 cells, there were no significant changes between cells treated with 100 μg/ml of 7-IP with control and untreated groups (Figure 3 D-F).

### 7-IP induces apoptosis in 5637 cells

**Figure 4:**
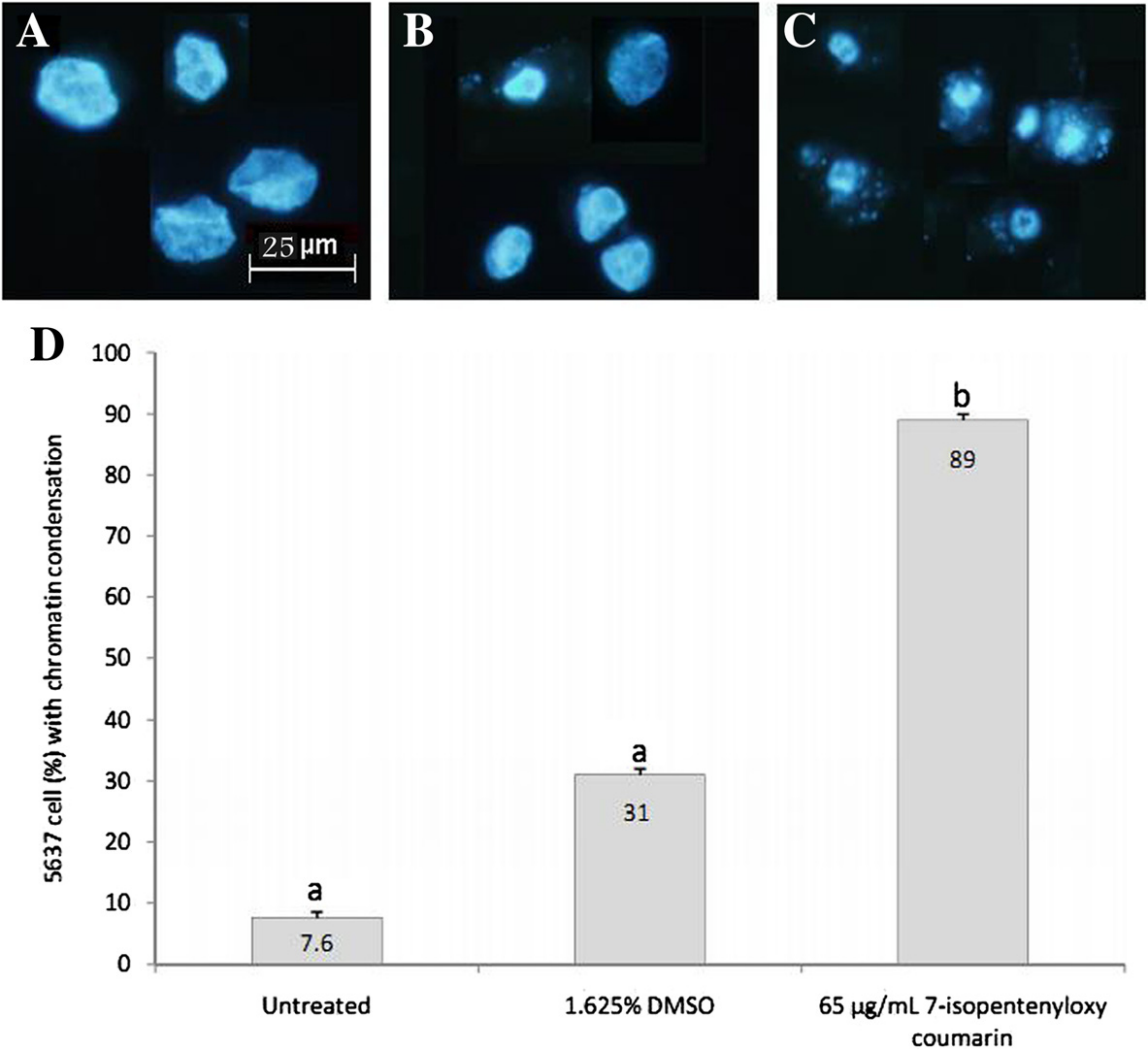
**Investigating the apoptotic morphology of cells by DAPI staining.** Chromatin condensation of 5637 cells without any treatment **(A)**, treated with 1.625% DMSO **(B)** and treated with 65 μg/ml 7-IP **(C)** for 72 h. The percentages of condensed chromatin, 72 h after treatments are compared with untreated and control groups **(D)**. Data are expressed as mean ± SD. The assay was done three times. Distinct letters indicate significant differences (*P* < 0.001) between cells treated with 65 μg/ml 7-IP and other cultures.

To determine what kind of cell death has been induced by 7-IP, the chromatin condensation and DNA damage were assessed using DAPI staining and alkaline comet assay, respectively. Based on MTT results, 5637 cells were treated with 65 μg/ml 7-IP and equivalent amount of DMSO for 72 h. As shown in Figure 4 (A-C) cells treated with this compound, showed highly condensed chromatin and nuclear fragmentation in comparison to control and untreated cells. Quantitative results revealed that 89% of cells treated with 65 μg/ml 7-IP presented condensed chromatin, which was significantly (*P* < 0.001) higher than control (31%) and untreated (7.5%) cultures (Figure 4D).

**Figure 5:**
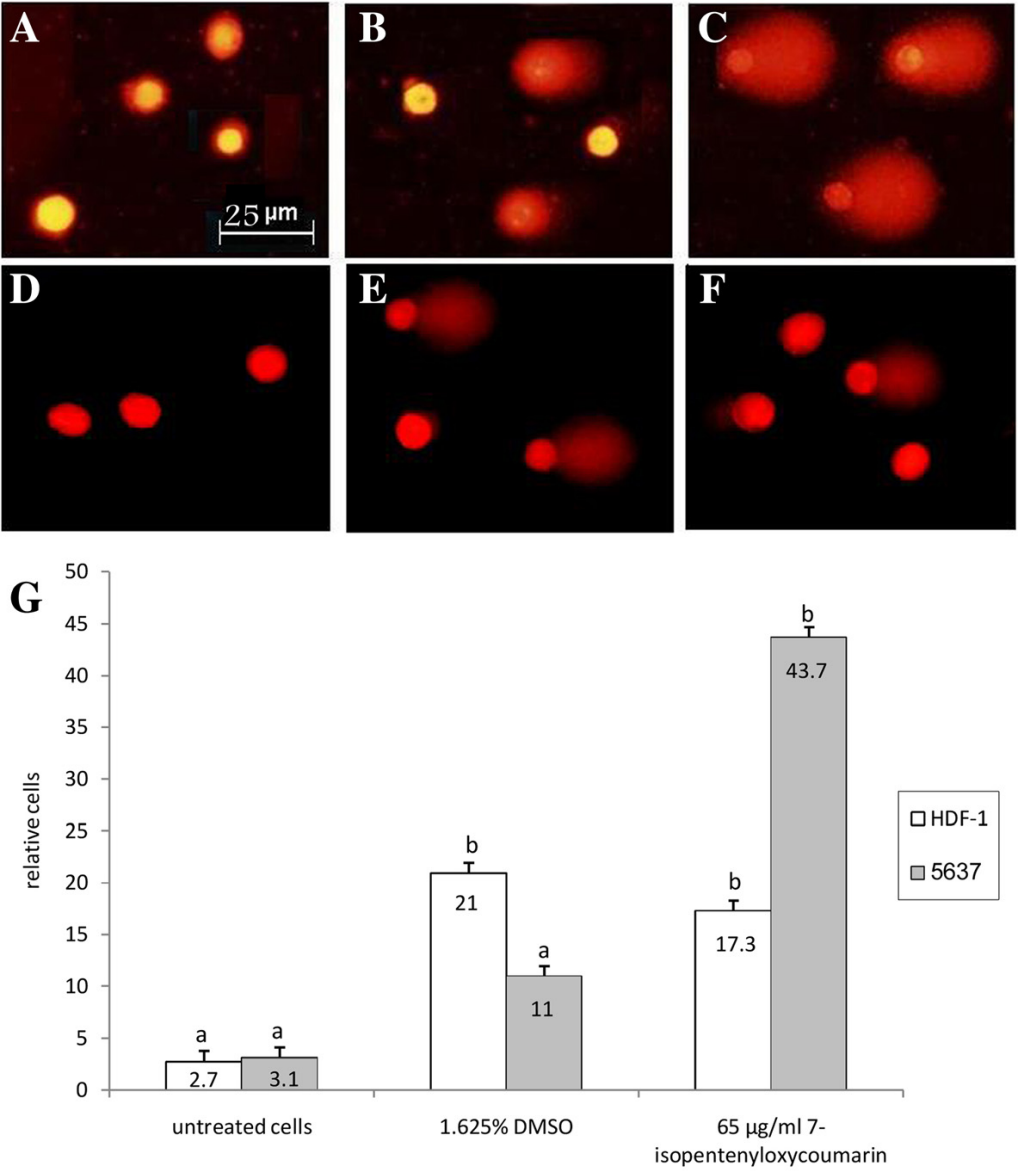
**Evaluating the DNA damaging effects of 7-IP by comet assay.** DNA damage of 5637 and HDF-1 cells without any treatment **(A and D)**, treated with 1.625% DMSO **(B and E)** and treated with 65 μg/ml 7-IP **(C and F)** for 72 h, respectively. The percentages of damaged DNA, 72 h after treatments are compared with untreated and control groups in both 5637 and HDF-1 cells **(G)**. Data are expressed as mean ± SD. The assay was repeated three times. Distinct letters show significant differences (*P* < 0.001) between different groups.

Furthermore, detecting DNA damage inducing effects of 7-IP in 5637 cells by comet assay, showed comet tail in a high percentage of individual cells treated with this coumarin (Figure 5 A-C). Data analysis showed that 7-IP induced approximately 43% DNA damage, significantly (*P* < 0.001) higher than that induced by its DMSO control (11%, Figure 5G). In order to investigate the genotoxic effects of 7-IP in non-cancerous cells, comet assay was also performed in HDF-1 cells treated with the same amount of the compound. As shown in Figure 5 (D-F) the difference between comet tail in HDF-1 and 5637 cells was obvious, treated HDF-1 cells represented a very small comet tail (Figure 5F). Statistical analysis revealed that the effect of 7-IP to induce DNA damage in HDF-1 cells (17%) is probably due to the presence of DMSO as a solvent since the DMSO control group induced the DNA damage by 21% (Figure 5G).

### 7-IP activates caspase-3

**Figure 6:**
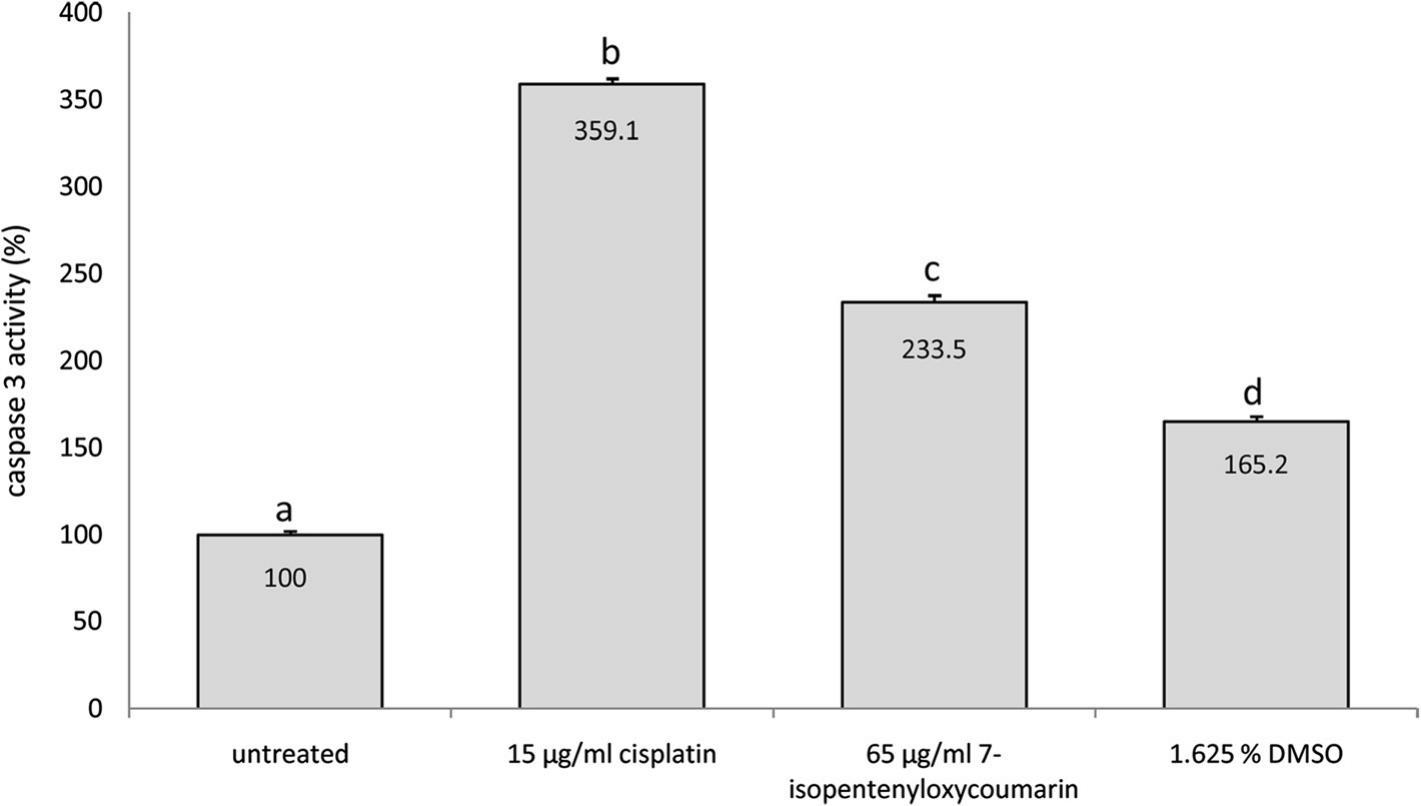
**The effects of 7-IP on caspase-3 activity in 5637 cells as detected by colorimetric assay.** 7-IP-treated cells show approximately 2.5 folds higher caspase activity than untreated cells. Cells treated with 15 μg/ml cisplatin showed elevated caspase-3 activity by approximately 3.5 folds. Data are expressed as mean ± SD. Results are from three independent experiments in triplicate. Distinct letters indicate significant differences (*P* < 0.001) between different groups.

To confirm the apoptotic effects of 7-IP, the activity of caspase-3 enzyme was also analyzed. After 12 h of incubation, the caspase activity in 7-IP-treated 5637 cells was increased approximately 2.5 folds as compared with untreated culture (Figure 6), significantly (*P* < 0.001) higher than DMSO-treated group.

### 7-IP arrests 5637 cells at G2/M stage

**Figure 7:**
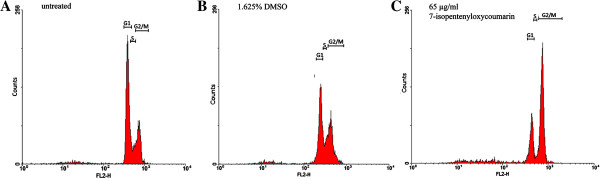
**G2/M arrest of 5637 cells treated with 7-IP, 24 h after treatment.** Cell cycle distribution of 5637 cells without any treatment **(A)**. Cells treated with 1.625% DMSO **(B)** do not show remarkable changes in cell cycle phases. Cells treated with 65 μg/ml 7-IP **(C)** show a marked cell cycle arrest at G2/M stage, 24 h after treatment.

To further study the anti-proliferative properties of 7-IP, its effects on cell cycle progression were evaluated by flow cytometry after PI staining. 5637 cells were treated with 65 μg/ml 7-IP and its equivalent amount of DMSO for 24 h. As shown in Figure 7, treatment with this compound caused an increase in the percentage of cells in G2/M correlating with decreased number of cells in G1 as compared with DMSO and untreated cultures. Thus, exposure to 7-IP led to cell cycle arrest at G2/M stage (Figure 7C). Percentages of cells at different stages of cell cycle are presented in Table 1.

**Table 1:** Effects of 7-IP on 5637 cell cycle distribution

**Treatment**	**Percentage of cells**		
	**G1 (Mean ± SD)**	**S (Mean ± SD)**	**G2/M (Mean ± SD)**
untreated	55.7 ± 6.16^a^	13.3 ± 1.20^a^	32.5 ± 0.88^a^
1.625% DMSO	43 ± 0.87^b^	12 ± 0.41^a^	36 ± 1.53^a^
65 μg/ml 7-IP	22.4 ± 1.63^c^	5.2 ± 1.47^b^	53.5 ± 2.54^b^

Percentages of cells in each stage of cell cycle is represented as mean ± SD. Results are from three independent experiments. Distinct letters in each column indicate statistical significant differences (*P* < 0.001) between groups.

## Discussion

Cancer is a consequence of unregulated cell growth with high morbidity and mortality worldwide. Despite major advances in cancer therapy, its treatment is far from being satisfactory and more research on new compounds and strategies to fight this disease is urgently needed.

As mentioned before, coumarins represent a large class of natural compounds with various biological activities, among which prenyloxycoumarins exert valuable anti-tumor properties both *in vitro* and *in vivo*[8,12,21].

In search for anti-cancer agents to treat bladder cancer, 7-IP was tested for its cytotoxicity on 5637 and HDF-1 cells (as control) *in vitro*. This compound belongs to prenyloxycoumarins with various biological properties, which can be synthesized both naturally and chemically [13]. Although this coumarin derivative exerts various biological properties, there is no report on its toxic effects and mechanism of action on bladder cancer cells in the literature. Our results revealed that the IC_50_ value of 7-IP in 5637 cells was 65 μg/ml (>100 μM) after 72 h of treatment, which is less than vinblastine but higher than vincristine and cisplatin [22]. Similar results were reported by Bruyere and coworkers who showed that 7-IP had IC_50_ values more than 100 μM in various human cancer cell lines [23]. Furthermore, Kawaii and colleagues determined low cytotoxicity of 7-IP on other cancers including lung, melanoma and leukemia [24]. On the other hand, Kofians and colleagues reported the ID_50_ values of 7-IP on KB and NSCLC-N6 cell lines, as 10.6 and 9.9 μg/ml, respectively [25]. Considering different reports on toxic effects of 7-IP, it can be concluded that depending on the cell line, the cytotoxicity of this coumarin would be different. High IC_50_ value of 7-IP in 5637 cells can be explained by the multi-drug resistance (MDR) of these cells [26,27]. Several ATP-binding cassette (ABC) transporters have been discovered, including multidrug resistance-associated proteins (MRPs) and P-glycoproteins, which play critical roles in MDR [28]. For instance, it has been reported that TCC cells over express MDR1 and MRP2 proteins, two members of MRP family [29]. Therefore, it is possible that 7-IP is exported from 5637 cells by these efflux pumps, so high amounts of this compound are required to be accumulated inside the cell, in order to induce cytotoxicity. To investigate the anti-cancer properties of 7-IP, non-cancerous HDF-1 cells were treated with the same concentrations as 5637 cells and results revealed no significant growth-inhibitory effects of this coumarin in HDF-1 cells (Figure 2B). This confirms the selective activity of 7-IP in cancerous 5637 cells.

Results of DAPI staining and comet assay also revealed that, in comparison with control cultures, 65 μg/ml 7-IP significantly (*P* < 0.001) increased both chromatin condensation and DNA damage in 5637 cells, which were in agreement with morphological observations and MTT results and indicate that cells are undergoing apoptotic cell death [30,31]. Moreover, to evaluate genotoxic effects of 7-IP in HDF-1 cells, comet assay was also performed in these cells. Comet results revealed that the DNA damaging effects of 7-IP were due to its solvent DMSO and there was no significant difference (*P* < 0.001) between these two groups. Thus, 7-IP does not have any significant toxic or genotoxic effects on normal HDF-1 cells.

To confirm the type of cell death, caspase-3 activity (the executioner upon which many apoptotic pathways converge) was evaluated using caspase-3 colorimetric assay kit. As shown in Figure 6, caspase activity in 7-IP-treated 5637 cells was elevated. The relative caspase-3 activity was increased about 2.5 folds in cells treated with this compound in comparison to untreated cells; which was also significantly increased compared to cells treated with equivalent amount of DMSO (*P* < 0.001).

Cell cycle analysis was performed by flow cytometry after PI staining to investigate the effects of 7-IP in more details. It was shown that this compound arrested 5637 cells in G2/M stage of the cell cycle (Figure 7C), which is similar to the effects of other anti-cancer drugs like taxol, doxorubicin and vincristine [32-34]. It is well established that progression of cell cycle is a tightly ordered and regulated process involving multiple checkpoints. Upregulation of cyclin B1/Cdc2 kinase activity, which regulates the entry and progression of the mitotic phase in eukaryotic cells, is known to be involved in the G2/M stage transition of the cell cycle. On the other hand, cells are arrested in M stage when microtubule network is disrupted [35]. Since 7-IP arrests 5637 cells in G2/M stage of the cell cycle, it is possible that this compound may act on cell cycle checkpoints or acts like a microtubule inhibitor, preventing further cell division and subsequently initiates cell death by apoptosis. Our results are similar to other studies that determined the ability of different coumarins in caspase-3 activation and cell cycle arrest. For instance, Barthomeuf and colleagues showed that umbelliprenin (another prenyloxycoumarin with a farnesyl chain) markedly inhibited proliferation of M4Beu cells through induction of caspase-dependent apoptosis and cell-cycle arrest [9]. Another study by Chuang and his coworkers revealed that coumarin induced cell cycle arrest and caspase-3 dependent apoptosis in HeLa cells [10].

## Conclusion

In summary, it can be concluded that 7-IP had toxic effects on 5637 cells. Exploring the anti-tumor activity of 7-IP showed that this coumarin has selective cytotoxic effects on 5637 cancerous cells in comparison to normal HDF-1 cells. Further studies in 5637 cells revealed that 7-IP induces chromatin condensation, DNA damage and apoptosis most probably via activation of caspase-3 and arrests the cell cycle at G2/M stage. Further studies are needed to determine its exact mechanism of action. For example, since antioxidants have different effects in cancer treatment [36], it is important to explore this property of 7-IP and its mechanism of action. Moreover, since *in vitro* conditions are very different from *in vivo* environments, in order to evaluate 7-IP effects on biological systems, an approved method on animal models is required [37].

Our study represents the first report describing 7-IP as an anti-tumor agent for bladder cancer cells *in vitro*. Although 7-IP was synthesized in this study, but considering the fact that it is widespread in edible vegetables and fruits, the present study could be regarded as a topic for future studies aiming to put in evidence dietary feeding chemopreventive effects on baldder cancer.

## Abbreviations

DAPI: 4′, 6-diamidino-2-phenylindole dichloride; DMEM: Dulbecco’s modified Eagle’s medium; DMSO: Dimethyl sulfoxide; EDTA: Ethylenediaminetetraacetic acid; ELISA: Enzyme linked immunosorbent assay; FBS: Fetal bovine serum; HDF1: Human dermal fibroblast 1; IC50: Half maximal inhibitory concentration; 7-IP: 7-isopentenyloxycoumarin; LMA: Low melting agarose; MTT: 3-(4,5-Dimethyl-2-thiazolyl)-2,5-diphenyl-2*H*-tetrazolium bromide; NMA: Normal melting agarose; NMR: Nuclear magnetic resonance; PBS: Phosphate buffered saline; PI: Propidium iodide; SD: Standard deviation; TCC: Transitional cell carcinoma.

## Competing interests

The authors have no conflict of interests to declare.

## Authors’ contributions

MMM conceived the strategy of study and supervised the project. FH performed the experimental work and data interpretation. ARB gave consultation on designing the study, complemented the data. MI provided the compound and gave consultation. FBR and AH were involved in performing the experimental work and data interpretation. All authors read and approved the final manuscript.

## Authors’ information

Fereshteh Haghighi, M.Sc. in Cell and Molecular Biology; Maryam M. Matin, Ph.D. in Molecular Biotechnology and Associate Professor at Ferdowsi University of Mashhad; Ahmad Reza Bahrami, Ph.D. in Molecular Biotechnology, Head of Institute of Biotechnology and Professor at Ferdowsi University of Mashhad; Mehrdad Iranshahi, Ph.D. in Pharmacognosy and Associate Professor at Mashhad University of Medical Sciences, Fatemeh B. Rassouli, Ph.D. in Cell and Molecular Biology and Azadeh Haghighitalab, M.Sc. in Cell and Molecular Biology.

## Supplementary Material

Additional file 1: Table S1 1H-NMR data for 7-isopentenyloxycoumarin (CDCl3, 500 MHz). Click here for file

Additional file 2: Table S2 13 C-NMR data for 7-isopentenyloxycoumarin (CDCl3, 125.7 MHz). Click here for file
