# Supplementary material for: The cytotoxic activities of 7-isopentenyloxycoumarin on 5637 cells via induction of apoptosis and cell cycle arrest in G2/M stage
Source: Daru. 2014 Jan 6;22(1):3. doi: 10.1186/2008-2231-22-3 (PMC3898598; doi:10.1186/2008-2231-22-3)
Supplement: Additional file 1: Table S1 — 1H-NMR data for 7-isopentenyloxycoumarin (CDCl3, 500 MHz). [file 2008-2231-22-3-S1.docx]

**Table S1:** 1H-NMR data for 7-isopentenyloxycoumarin (CDCl3, 500 MHz) ^a^

| **H** | **7-isopentenyloxycoumarin** |
| --- | --- |
| 2  3  4  5  6  7  8  9  10  1'  2'  3'  4'  5'  6'  7'  8'  9'  10'  11'  12'  13'  14'  15' | -  6.24 d (9.6)  7.63 d (9.6)  7.36 d (7.2)  6.85 dd (7.2, 2.0)  -  6.82 d (2.0)  -  -  4.57 d (7.0)  5.47 t (7.0)  -  1.80 m  1.76 m  -  -  -  -  -  -  -  -  -  - |

^a^ J values are in parenthesis and reported in Hz; chemical shifts are given in ppm
