# Supplementary material for: The cytotoxic activities of 7-isopentenyloxycoumarin on 5637 cells via induction of apoptosis and cell cycle arrest in G2/M stage
Source: Daru. 2014 Jan 6;22(1):3. doi: 10.1186/2008-2231-22-3 (PMC3898598; doi:10.1186/2008-2231-22-3)
Supplement: Additional file 2: Table S2 — 13 C-NMR data for 7-isopentenyloxycoumarin (CDCl3, 125.7 MHz). [file 2008-2231-22-3-S2.doc]

**Table S2:** 13 C-NMR data for 7-isopentenyloxycoumarin (CDCl3, 125.7 MHz)

| **C** | **7-isopentenyloxycoumarin** |
| --- | --- |
| 2  3  4  5  6  7  8  9  10  1'  2'  3'  4'  5'  6'  7'  8'  9'  10'  11'  12'  13'  14'  15' | 161.2  112.9  143.4  128.6  113.1  162.0  101.5  155.8  112.3  65.3  118.5  139.2  25.7  18.2  -  -  -  -  -  -  -  -  -  - |
